# Supplementary material for: Shifting from fear to safety through deconditioning-update
Source: eLife. 2020 Jan 30;9:e51207. doi: 10.7554/eLife.51207 (PMC7021486; doi:10.7554/eLife.51207)
Supplement: Supplementary file 1. [file elife-51207-supp1.docx]

**Table 1.** **Weakening fear memory through deconditioning-update training**

| **Figure 1** | | | | | | |
| --- | --- | --- | --- | --- | --- | --- |
| Figure 1B. Reactivations | | | | | | |
| Omnibus test | | η² | *P* value | Post-hoc (Bonferroni) | | *P* value |
| Two-way RM ANOVA | Interaction  F_(3,33)_ = 5.897  Time  F_(3,33)_ = 37.1  Group  F_(1,11)_ = 20.24 | 0.87  0.55  0.15 | 0.002  < 0.0001  0.0009 | Day 3  Day 4  Day 5  Day 6 | | 0.99  0.99  0.001  0.0001 |
| Figure 1C. Test | | | | | | |
| Omnibus Test | | η² | *P* value | Post-hoc (Tukey) | | *P* value |
| One-way ANOVA | F_(2,17)_ = 19.57 | 0.7 | < 0.0001 | control vs. footshock  control vs. no-footshock  footshock vs. no-footshock | | < 0.0001  0.16  0.001 |
| Figure 1D. Renewal | | | | | | |
| Omnibus Test | | η² | *P* value | Post-hoc (Tukey) | | *P* value |
| One-way ANOVA | F_(2,17)_ = 33.41 | 0.8 | < 0.0001 | control vs. footshock  control vs. no-footshock  footshock vs. no-footshock | | < 0.0001  0.36  0.0001 |
| Figure 1E. Spontaneous Recovery | | | | | | |
| Omnibus Test | | η² | *P* value | Post-hoc (Tukey) | | *P* value |
| One-way ANOVA | F_(2,17)_ = 17.38 | 0.67 | < 0.0001 | control vs. footshock  control vs. no-footshock  footshock vs. no-footshock | | < 0.0001  0.2  0.002 |
| *N per group:*  Control = 7; Footshock = 6; No-footshock = 7 | | | | | | |
| Figure 1G. Reactivations | | | | | | |
| Omnibus Test | | η² | *P* value | Post-hoc (Bonferroni) | *P* value | |
| Two-way RM ANOVA | Interaction  F_(3,36)_ = 1.556  Time  F_(3,36)_ = 38.52  Group  F_(1,12)_ = 3.598 | 0.18  0.45  0.90 | 0.22  < 0.0001  0.08 | Day 3  Day 4  Day 5  Day 6 | | 0.99  0.13  0.12  0.9 |
| Figure 1H. Test | | | | | | |
| Omnibus Test | | η² | *P* value | Post-hoc (Tukey) | | *P* value |
| One-way ANOVA | F_(2,17)_ = 16.82 | 0.66 | < 0.0001 | control vs. footshock  control vs. no-footshock  footshock vs. no-footshock | | < 0.0001  0.009  0.05 |
| Figure 1I. Renewal | | | | | | |
| Omnibus Test | | η² | *P* value | Post-hoc (Tukey) | | *P* value |
| One-way ANOVA | F_(2,17)_ = 43.69 | 0.84 | < 0.0001 | control vs. footshock  control vs. no-footshock  footshock vs. no-footshock | | 0.0001  0.007  0.0001 |
| Figure 1J. Spontaneous Recovery | | | | | | |
| Omnibus Test | | η² | *P* value | Post-hoc (Tukey) | | *P* value |
| One-way ANOVA | F_(2,17)_ = 40.37 | 0.83 | < 0.0001 | control vs. footshock  control vs. no-footshock  footshock vs. no-footshock | | < 0.0001  0.02  < 0.0001 |
| Figure 1K. Retraining | | | | | | |
| Omnibus Test | | η² | *P* value | Post hoc (Tukey) | | *P* value |
| One-way ANOVA | F_(2,17)_ = 29.02 | 0.77 | < 0.0001 | control vs. footshock  control vs. no-footshock  footshock vs. no-footshock | | < 0.0001  0.1  0.0002 |
| *N per group:*  Control = 6; Footshock = 7; No-footshock = 7 | | | | | | |

RM – repeated measures; ANOVA – Analysis of Variance.
