# Supplementary material for: Shifting from fear to safety through deconditioning-update
Source: eLife. 2020 Jan 30;9:e51207. doi: 10.7554/eLife.51207 (PMC7021486; doi:10.7554/eLife.51207)
Supplement: Supplementary file 2. [file elife-51207-supp2.docx]

**Table 2.** **Deconditioning-update approach weakens both remote and strong fear memory**.

| **Figure 2** | | | | | |
| --- | --- | --- | --- | --- | --- |
| Figure 2B. Reactivations | | | | | |
| Omnibus test | | η² | *P* value | Post-hoc (Bonferroni) | *P* value |
| Two-way RM ANOVA | Interaction  F_(3,33)_ = 4.959  Time  F_(3,33)_ = 22.72  Group  F_(1,11)_ = 13.08 | 0.10  0.41  0.17 | 0.006  0.0001  0,004 | Day 3  Day 4  Day 5  Day 6 | 0.99  0.33  0.001  0.0009 |
| Figure 2C. Test | | | | | |
| Omnibus Test | | η² | *P* value | Post-hoc (Tukey) | *P* value |
| One-way ANOVA | F_(2,15)_ = 11.63 | 0.61 | 0.0009 | control vs. footshock  control vs. no-footshock  footshock vs. no-footshock | 0.002  0.002  0.9 |
| Figure 2D. Renewal | | | | | |
| Omnibus Test | | η² | *P* value | Post-hoc (Tukey) | *P* value |
| One-way ANOVA | F_(2,15)_ = 31.06 | 0.81 | 0.0001 | control vs. footshock  control vs. no-footshock  footshock vs. no-footshock | 0.0001  0.017  0.0004 |
| Figure 2E. Spontaneous Recovery | | | | | |
| Omnibus Test | | R^2^ | *P* value | Post-hoc (Tukey) | *P* value |
| One-way ANOVA | F_(2,15)_ = 21.68 | 0.74 | 0.0001 | control vs. footshock  control vs. no-footshock  footshock vs. no-footshock | 0.0001  0.06  0.001 |
| *N per group:*  Control = 5; Footshock = 6; No-footshock = 7 | | | | | |
| Figure 2G. Reactivations | | | | | |
| Omnibus Test | | η² | *P* value | Post-hoc (Bonferroni) | *P* value |
| Two-way RM ANOVA | Interaction  F_(3,36)_ = 7.596  Time  F_(3,36)_ = 27.23  Group  F_(1,12)_ = 4.304 | 0.10  0.36  0.10 | 0.0005  0.0001  0.06 | Day 3  Day 4  Day 5  Day 6 | 0.99  0.99  0.003  0.015 |
| Figure 2H. Test | | | | | |
| Omnibus Test | | η² | *P* value | Post-hoc (Tukey) | *P* value |
| One-way ANOVA | F_(2,17)_ = 27.83 | 0.77 | 0.0001 | control vs. footshock  control vs. no-footshock  footshock vs. no-footshock | < 0.0001  0.004  0.004 |
| Figure 2I. Renewal | | | | | |
| Omnibus Test | | η² | *P* value | Post-hoc (Tukey) | *P* value |
| One-way ANOVA | F_(2,17)_ = 10.24 | 0.55 | 0.0012 | control vs. footshock  control vs. no-footshock  footshock vs. no-footshock | 0.002  0.87  0.004 |
| Figure 2J. Spontaneous Recovery | | | | | |
| Omnibus Test | | η² | *P* value | Post-hoc (Tukey) | *P* value |
| One-way ANOVA | F_(2,17)_ = 11.62 | 0.58 | 0.0007 | control vs. footshock  control vs. no-footshock  footshock vs. no-footshock | 0.001  0.8  0.003 |
| *N per group:*  Control = 6; Footshock = 7; No-footshock = 7 | | | | | |
