# Supplementary material for: Shifting from fear to safety through deconditioning-update
Source: eLife. 2020 Jan 30;9:e51207. doi: 10.7554/eLife.51207 (PMC7021486; doi:10.7554/eLife.51207)
Supplement: Supplementary file 3. [file elife-51207-supp3.docx]

**Table 3.** **Deconditioning-updating weakens fear memory in different behavioral tasks**.

| **Figure 3** | | | | | |
| --- | --- | --- | --- | --- | --- |
| Figure 3B. Reactivations | | | | | |
| Omnibus Test | | η² | *P* value | Post-hoc (Bonferroni) | *P* value |
| Two-way RM ANOVA | Interaction  F_(3,54)_ = 3.516  Time  F_(3,54)_ = 37.87  Group  F_(1,18)_ = 9.109 | 0.03  0.32  0.17 | 0.02  0.0001  0.007 | Day 3  Day 4  Day 5  Day 6 | 0.99  0.12  0.003  0.004 |
| Figure 3C. Test | | | | | |
| Omnibus Test | | η² | *P* value | Post-hoc (Tukey) | *P* value |
| One-way ANOVA | F_(2,25)_ = 19.76 | 0.61 | 0.0001 | control vs. footshock  control vs. no-footshock  footshock vs. no-footshock | 0.002  0.002  0.9 |
| Figure 3D. Spontaneous Recovery | | | | | |
| Omnibus Test | | η² | *P* value | Post-hoc (Tukey) | *P* value |
| One-way ANOVA | F_(2,25)_ = 6.370 | 0.34 | 0.005 | control vs. footshock  control vs. no-footshock  footshock vs. no-footshock | 0.00010.06  0.001 |
| *N per group:*  Control = 8; Footshock = 10; No-footshock = 10 | | | | | |
| Figure 3F. Test | | | | | |
| Omnibus Test | | η² | *P* value | Post hoc (Dunn) | *P* value |
| Kruskal-Wallis | H = 13.96 | 0.48 | 0.0009 | control vs. footshock  control vs. no-footshock  footshock vs. no-footshock | 0.001  0.73  0.02 |
| Figure 3G. Test | | | | | |
| Omnibus Test | | η² | *P* value | Post-hoc (Dunn) | *P* value |
| Kruskal-Wallis | H = 17.03 | 0.60 | 0.0002 | control vs. footshock  control vs. no-footshock  footshock vs. no-footshock | 0.001  0.99  0.0009 |
| *N per group:*  Control = 8; Footshock = 10; No-footshock = 10 | | | | | |
