# Supplementary material for: Shifting from fear to safety through deconditioning-update
Source: eLife. 2020 Jan 30;9:e51207. doi: 10.7554/eLife.51207 (PMC7021486; doi:10.7554/eLife.51207)
Supplement: Supplementary file 4. [file elife-51207-supp4.docx]

**Table 4.** **Deconditioning-update is based on memory destabilization mechanisms.**

| **Figure 4** | | | | | | | | |
| --- | --- | --- | --- | --- | --- | --- | --- | --- |
| Figure 4B. Extinction Sessions | | | | | | | | |
| Omnibus Test | | | η² | | *P* value | | Post-hoc (Bonferroni) | *P* value |
| Two-way RM ANOVA | Interaction  F_(11,132)_ = 8.041  Time  F_(11,132)_ = 17.16  Group  F_(1,12)_ = 19.65 | | 0.12  0.27  0.26 | | < 0.0001  < 0.0001  0.0008 | | T1+T2  T3+T4  T5+T6  T7+T8  T9+T10  T11+T12  T13+T14  T15+T16  T17+T18  T19+T20  T21+T22  T23+T24 | 0.99  0.99  0.99  0.46  0.37  0.0002 0.002  0.001  0.21  0.0001  0.0001  0.0001 |
| Figure 4C. Test | | | | | | | | |
| Omnibus Test | | η² | | *P* value | | Post-hoc (Tukey) | | *P* value |
| One-way ANOVA | F_(2,17)_ = 46.37 | 0.85 | | 0.0001 | | control vs. footshock  control vs. no-footshock  footshock vs. no-footshock | | 0.002  0.002  0.9 |
| Figure 4D. Renewal | | | | | | | | |
| Omnibus Test | | η² | | *P* value | | Post-hoc (Tukey) | | *P* value |
| One-way ANOVA | F_(2,17)_ = 2.453 | 0.22 | | 0.11 | | control vs. footshock  control vs. no-footshock  footshock vs. no-footshock | | 0.0001  0.017  0.0004 |
| Figure 4E. Spontaneous Recovery | | | | | | | | |
| Omnibus Test | | η² | | *P* value | | Post-hoc (Tukey) | | *P* value |
| One-way ANOVA | F_(2,17)_ = 5.668 | 0.4 | | 0.01 | | control vs. footshock  control vs. no-footshock  footshock vs. no-footshock | | 0.0001  0.06  0.001 |
| *N per group:*  Control = 6; Footshock = 7; No-footshock = 7 | | | | | | | | |
| Figure 4G. Reactivations | | | | | | | | |
| Omnibus Test | | η² | | *P* value | | Post-hoc (Bonferroni) | | *P* value |
| Three-way RM ANOVA | Time  F_(3,78)_ = 47.9  Drug  F_(1,26)_ = 16.46  Footshock  F_(1,25)_ = 0.1236  Time x Drug  F_(3,78)_ = 13.36  Time x Footshock  F_(3,78)_ = 0.2317  Drug x Footshock  F_(1,26)_ = 1.431  3-way Interaction  F_(3,78)_ = 1.021 | 0.31  0.16  0.001  0.09  0.002  0.01  0.01 | | 0.0004  < 0.0001  0.73  <0.0001  0.87  0.24  0.39 | | Day 3  NFS Veh vs. NFS Nimo  FS Veh vs. FS Nimo  NFS Veh vs. FS Veh  NFS Nimo vs. FS Nimo  Day 4  NFS Veh vs. NFS Nimo  FS Veh vs. FS Nimo  NFS Veh vs. FS Veh  NFS Nimo vs. FS Nimo  Day 5  NFS Veh vs. NFS Nimo  FS Veh vs. FS Nimo  NFS Veh vs. FS Veh  NFS Nimo vs. FS Nimo  Day 6  NFS Veh vs. NFS Nimo  FS Veh vs. FS Nimo  NFS Veh vs. FS Veh  NFS Nimo vs. FS Nimo | | >0.99  >0.99  >0.99  >0.99  >0.99  0.001  >0.99  >0.99  0.49  0.07  >0.99  >0.99  >0.99  0.01  >0.99  >0.99 |
| Figure 4H. Test | | | | | | | | |
| Omnibus Test | | η² | | *P* value | | Post-hoc (Tukey) | | *P* value |
| Two-way RM ANOVA | Interaction  F_(1,25)_ = 0.7442  Drug  F_(1,25)_ = 7.890  Footshock  F_(1,25)_ = 0.9 | 0.02  0.23  0.03 | | 0.39  0.009  0.35 | | Tukey’s  Nimo NFS vs. Nimo FS  Nimo NFS vs. Veh NFS  Nimo NFS vs. Veh FS  Nimo FS vs. Veh NFS  Nimo FS vs. Veh FS  Veh NFS vs. Veh FS | | 0.99  0.53  0.06  0.55  0.06  0.59 |
| Figure 4I. Renewal | | | | | | | | |
| Omnibus Test | | η² | | *P* value | | Post-hoc (Tukey) | | *P* value |
| Two-way RM ANOVA | Interaction  F_(1,25)_ = 10.34  Drug  F_(1,25)_ = 19.11  Group  F_(1,25)_ = 7.239 | 0.17  0.31  0.12 | | 0.003  0.0002  0.01 | | Tukey’s  Nimo NFS vs. Nimo FS  Nimo NFS vs. Veh NFS  Nimo NFS vs. Veh FS  Nimo FS vs. Veh NFS  Nimo FS vs. Veh FS  Veh NFS vs. Veh FS | | 0.98  0.85  0.0003  0.63  < 0.0001  0.002 |
| Figure 4J. Spontaneous Recovery | | | | | | | | |
| Omnibus Test | | η² | | *P* value | | Post hoc (Tukey) | | *P* value |
| Two-way RM ANOVA | Interaction  F_(1,25)_ = 3.525  Drug  F_(1,25)_ = 9.11  Group  F_(1,25)_ = 6.349 | 0.08  0.21  0.14 | | 0.07  0.005  0.01 | | Tukey’s  Nimo NFS vs. Nimo FS  Nimo NFS vs. Veh NFS  Nimo NFS vs. Veh FS  Nimo FS vs. Veh NFS  Nimo FS vs. Veh FS  Veh NFS vs. Veh FS | | 0.97  0.84  0.003  0.98  0.008  0.02 |
| *N per group:*  No-footshock vehicle = 7; No-footshock nimodipine = 7; Footshock vehicle =7; Footshock nimodipine = 8 | | | | | | | | |

Nimo – nimopidine; NFS – no-footshock; FS – footshock
