# Supplementary material for: Shifting from fear to safety through deconditioning-update
Source: eLife. 2020 Jan 30;9:e51207. doi: 10.7554/eLife.51207 (PMC7021486; doi:10.7554/eLife.51207)
Supplement: Supplementary file 5. [file elife-51207-supp5.docx]

**Table 5. Deconditioning-update does not occur with 0.3-mA shocks.**

| **Figure 1-figure supplement 1** | | | | | |
| --- | --- | --- | --- | --- | --- |
| Figure 1S1B. Reactivations | | | | | |
| Omnibus Test | | η² | *P* value | Post-hoc (Bonferroni) | *P* value |
| Two-way RM ANOVA | Interaction  F_(3,36)_ = 6.610  Time  F_(3,36)_ = 11.51  Group  F_(1,12)_ = 2.922 | 0.10  0.17  0.11 | 0.001  < 0.0001  0.11 | Day 3  Day 4  Day 5  Day 6 | > 0.99  > 0.99  0.11  0.01 |
| Figure 1S1C. Test | | | | | |
| Omnibus Test | | η² | *P* value | Post-hoc (Tukey) | *P* value |
| One-way ANOVA | F_(2,17)_ = 8.889 | 0.49 | 0.0023 | control vs. footshock  control vs. no-footshock  footshock vs. no-footshock | 0.81  0.003  0.009 |
| *N per group:*  Control = 6; No-footshock = 7; Footshock = 7 | | | | | |
