# Supplementary material for: Shifting from fear to safety through deconditioning-update
Source: eLife. 2020 Jan 30;9:e51207. doi: 10.7554/eLife.51207 (PMC7021486; doi:10.7554/eLife.51207)
Supplement: Supplementary file 6. [file elife-51207-supp6.docx]

**Table 6. A single reactivation session does not update fear memory.**

| **Figure 1-figure supplement 2** | | | |
| --- | --- | --- | --- |
| Figure 1S2B. Reactivation | | | |
| Omnibus Test | | η² | *P* value |
| Student's *t* test | T_12_ = 1.440 | 0.15 | 0.17 |
| Figure 1S2C. Test | | | |
| Omnibus Test | | η² | *P* value |
| One-way ANOVA | F_(2,17)_ = 2.694 | 0.24 | 0.1 |
| Figure 1S2C. Renewal | | | |
| Omnibus Test | | η² | *P* value |
| One-way ANOVA | F_(2,17)_ = 0.7905 | 0.21 | 0.47 |
| *N per group:*  Control = 6; No-footshock = 7; Footshock = 7 | | | |
