# Supplementary material for: Shifting from fear to safety through deconditioning-update
Source: eLife. 2020 Jan 30;9:e51207. doi: 10.7554/eLife.51207 (PMC7021486; doi:10.7554/eLife.51207)
Supplement: Supplementary file 7. [file elife-51207-supp7.docx]

**Table 7. Deconditioning-update is not due to US devaluation**.

| **Figure 1-figure supplement 3** | | | | | | | |
| --- | --- | --- | --- | --- | --- | --- | --- |
| Figure 1S3B. Test | | | | | | | |
| Omnibus Test | | | η² | *P* value | Post-hoc (Tukey) | | *P* value |
| One-way ANOVA | F_(2,18)_ = 23.16 | | 0.72 | < 0.0001 | control vs. footshock  control vs. devaluation  footshock vs. devaluation | | < 0.0001  0.551  0.0001 |
| *N per group:*  Control = 7; No-footshock = 7; Footshock = 7 | | | | | | | |
| Figure 1S3D.Reactivations | | | | | | | |
| Omnibus Test | | | η² | *P* value | Post-hoc (Bonferroni) | | *P* value |
| Two-way RM ANOVA | Interaction  F_(3,54)_ = 0.06901  Time  F_(3,54)_ = 21.81  Group  F_(1,18)_ = 0.03687 | | 0.001  0.34  0.0007 | 0.976  < 0.0001  0.849 | Day 1  Day 2  Day 3  Day 4 | | > 0.99  > 0.99  > 0.99  > 0.99 |
| Figure 1S3E. Test | | | | | | | |
| Test | | | | R^2^ | | *P* value | |
| Student's *t* test | | T_18_ = 0.9815 | | 0.05 | | 0.33 | |
| Figure 1S3E. Reinstatement | | | | | | | |
| Test | | | | R^2^ | | *P* value | |
| Student's *t* test | | T_18_ = 3.102 | | 0.35 | | 0.006 | |
| *N per group:*  No-footshock = 10; Footshock = 10 | | | | | | | |
