# Supplementary material for: Shifting from fear to safety through deconditioning-update
Source: eLife. 2020 Jan 30;9:e51207. doi: 10.7554/eLife.51207 (PMC7021486; doi:10.7554/eLife.51207)
Supplement: Supplementary file 8. [file elife-51207-supp8.docx]

**Table 8. Deconditioning-update does not occur with unpaired shocks in the reactivation sessions.**

| **Figure 1-figure supplement 4** | | | | | | |
| --- | --- | --- | --- | --- | --- | --- |
| Figure 1S4B. Reactivations | | | | | | |
| Omnibus test | | η² | *P* value | Post-hoc (Bonferroni) | | *P* value |
| Two-way RM ANOVA | Interaction  F_(3,42)_ = 32.01  Time  F_(3,42)_ = 27.42  Group  F_(1,14)_ = 12.1 | 0.34  0.28  0.10 | 0.03  < 0.0001  0.0037 | Day 3  Day 4  Day 5  Day 6 | | > 0.99  > 0.99  > 0.99  < 0.0001 |
| Figure 1S4C. Test | | | | | | |
| Omnibus Test | | | | R^2^ | *P* value | |
| Student’s *t* test | | T_14_ = 5 | | 0.64 | 0.0002 | |
| *N per group:*  Footshock = 8; Footshock CS-US = 8 | | | | | | |
