# Supplementary material for: Shifting from fear to safety through deconditioning-update
Source: eLife. 2020 Jan 30;9:e51207. doi: 10.7554/eLife.51207 (PMC7021486; doi:10.7554/eLife.51207)
Supplement: Supplementary file 9. [file elife-51207-supp9.docx]

**Table 9. Deconditioning-update weakens strong fear memories in females.**

| **Figure 2-figure supplement 1** | | | | | |
| --- | --- | --- | --- | --- | --- |
| Figure 2S1B. Reactivations | | | | | |
| Omnibus test | | η² | *P* value | Post-hoc (Bonferroni) | *P* value |
| Two-way RM ANOVA | Interaction  F_(2,22)_ = 3.835  Time  F_(2,22)_ = 19.63  Group  F_(1,11)_ = 5.256 | 0.04  0.19  0.002 | 0.03  < 0.0001  0.04 | Day 3  Day 4  Day 5 | > 0.99  0.99  0.003 |
| Figure 2S1C. Test | | | | | |
| Omnibus Test | | η² | *P* value | Post-hoc (Tukey) | *P* value |
| One-way ANOVA | F_(2,16)_ = 22.02 | 0.73 | < 0.0001 | control vs. footshock  control vs. no-footshock  footshock vs. no-footshock | < 0.0001  0.01  0.005 |
| Figure 2S1C. Renewal | | | | | |
| Omnibus Test | | η² | *P* value | Post-hoc (Tukey) | *P* value |
| One-way ANOVA | F_(2,16)_ = 19.84 | 0.71 | < 0.0001 | control vs. footshock  control vs. no-footshock  footshock vs. no-footshock | < 0.0001  0.03  0.005 |
| *N per group:*  Control = 6; Footshock = 6; No-footshock = 7 | | | | | |
