# Supplementary material for: Shifting from fear to safety through deconditioning-update
Source: eLife. 2020 Jan 30;9:e51207. doi: 10.7554/eLife.51207 (PMC7021486; doi:10.7554/eLife.51207)
Supplement: Supplementary file 10. [file elife-51207-supp10.docx]

**Table 10. Deconditioning-update does not occur in a single 12-CS extinction session**.

| **Figure 4-figure supplement 1** | | | | | |
| --- | --- | --- | --- | --- | --- |
| Figure 4S1B. Extinction Session | | | | | |
| Omnibus test | | η² | *P* value | Post-hoc (Bonferroni) | *P* value |
| Two-way RM ANOVA | Interaction  F_(5,60)_ = 1.238  Time  F_(5,60)_ = 4.973  Group  F_(1,12)_ = 0.0946 | 0.05  0.19  0.002 | 0.3  0.0007  0.763 | T1+T2  T3+T4  T5+T6  T7+T8  T9+T10  T11+T12 | > 0.99  > 0.99  > 0.99  > 0.99  > 0.99  0.48 |
| Figure 4S1C. Test | | | | | |
| Omnibus Test | | η² | *P* value | Post-hoc | *P* value |
| One-way ANOVA | F_(2,17)_ = 1.079 | 0.09 | 0.36 | NA | NA |
| Figure 4S1C. Renewal | | | | | |
| Omnibus Test | | η² | *P* value | Post-hoc | *P* value |
| One-way ANOVA | F_(2,17)_ = 5.782 | 0.33 | 0.01 | control vs. footshock  control vs. no-footshock  footshock vs. no-footshock | 0.01  0.88  0.04 |
| Figure 4S1C. Spontaneous Recovery | | | | | |
| Omnibus Test | | R^2^ | *P* value | Post-hoc | *P* value |
| One-way ANOVA | F_(2,17)_ = 3.386 | 0.28 | 0.058 | NA | NA |
| *N per group:*  Control = 6; Footshock = 7; No-footshock = 7 | | | | | |
