# Supplementary material for: Shifting from fear to safety through deconditioning-update
Source: eLife. 2020 Jan 30;9:e51207. doi: 10.7554/eLife.51207 (PMC7021486; doi:10.7554/eLife.51207)
Supplement: Supplementary file 11. [file elife-51207-supp11.docx]

**Table 11. Nimodipine does not affect open field** **behavior and does not induce a state-dependent memory.**

| **Figure 4-figure supplement 2** | | | | | | |
| --- | --- | --- | --- | --- | --- | --- |
| Figure 4S2B. Reactivations | | | | | | |
| Omnibus test | | η² | *P* value | Post-hoc (Bonferroni) | | *P* value |
| Two-way RM ANOVA | Interaction  F_(3,54)_ = 5.8  Time  F_(3,54)_ = 105.6  Group  F_(1,18)_ = 8.411 | 0.04  0.72  0.04 | 0.0016  < 0.0001  0.009 | Day 3  Day 4  Day 5  Day 6 | | > 0.99  > 0.99  0.057  < 0.0001 |
| Figure 4S2C. Test 1 | | | | | | |
| Omnibus Test | | η² | *P* value | Post-hoc (Tukey) | | *P* value |
| One-way ANOVA | F_(2,17)_ = 21.22 | 0.71 | < 0.0001 | FS + V vs. FS + Nimo 1  FS + V vs. FS + Nimo 2  FS + Nimo 1 vs. FS + Nimo 2 | | 0.0003  < 0.0001  0.4928 |
| Figure 4S2C. Test 2 | | | | | | |
| Omnibus Test | | η² | *P* value | Post-hoc (Tukey) | | *P* value |
| One-way ANOVA | F_(2,16)_ = 19.84 | 0.69 | < 0.0001 | control vs. footshock  control vs. no-footshock  footshock vs. no-footshock | | < 0.0001  0.03  0.005 |
| *N per group:*  Footshock + Vehicle = 6; Footshock + Nimodipine 1 = 7; Footshock + Nimodipine 2 = 7 | | | | | | |
| Figure 4S2D. Test | | | | | | |
| Omnibus Test | | | | R^2^ | *P* value | |
| Student's *t* test | | T_18_ = 0.08669 | | 0.0004 | 0.93 | |
| Figure S7E. Test | | | | | | |
| Omnibus Test | | | | R^2^ | *P* value | |
| Student's *t* test | | T_18_ = 1.121 | | 0.07 | 0.27 | |
| *N per group:*  Vehicle = 10; Nimodipine = 10 | | | | | | |

Nimo – nimopidine; V – vehicle; FS – footshock
