# Supplementary material for: Shifting from fear to safety through deconditioning-update
Source: eLife. 2020 Jan 30;9:e51207. doi: 10.7554/eLife.51207 (PMC7021486; doi:10.7554/eLife.51207)
Supplement: Supplementary file 12. [file elife-51207-supp12.docx]

**Table 12. Baseline (pre-CS) freezing levels for Figure 1.**

| Figure 1 | |
| --- | --- |
| Reactivations | |
| Group | Baseline (% ± SEM) |
| Day 3  No Footshock  Footshock  Day 4  No Footshock  Footshock  Day 5  No Footshock  Footshock  Day 6  No Footshock  Footshock | 42.38 ± 13,58  42.22 ± 12.4  67.62 ± 8.15  37.22 ± 13.97  69.05 ± 12.17  61.67 ± 9.8  49.52 ± 10.75  45.56 ± 12.61 |
| Test | |
| Group | Baseline (% ± SEM) |
| Control  Footshock  No Footshock | 58.57 ± 10.26  7.22 ± 3.69  25.24 ± 7.41 |
| Renewal | |
| Group | Baseline (% ± SEM) |
| Control  Footshock  No Footshock | 64.29 ± 11.17  19.44 ± 11.62  25.24 ± 9.2 |
| Spontaneous Recovery | |
| Group | Baseline (% ± SEM) |
| Control  Footshock  No Footshock | 68.1 ± 9.56  35.56 ± 14.18  25.24 ± 9.2 |
| Reactivations | |
| Group | Baseline (% ± SEM) |
| Day 3  No Footshock  Footshock  Day 4  No Footshock  Footshock  Day 5  No Footshock  Footshock  Day 6  No Footshock  Footshock | 57.14 ± 11.09  20.95 ±12.72  32.86 ±16.5  2.86 ± 2.86  15.71 ± 10.2  10.48 ± 10.48  9.05 ± 7.99  0.95 ± 0.95 |
| Test | |
| Group | Baseline (% ± SEM) |
| Control  Footshock  No Footshock | 57.22 ± 16.34  0 ± 0  9.05 ± 9.05 |
| Renewal | |
| Group | Baseline (% ± SEM) |
| Control  Footshock  No Footshock | 52.78 ± 18.47  7.14 ± 4.96  27.62 ± 12.49 |
| Spontaneous Recovery | |
| Group | Baseline (% ± SEM) |
| Control  Footshock  No Footshock | 7.22 ± 7.22  0 ± 0  9.52 ± 11.3 |
| Retraining | |
| Group | Baseline (% ± SEM) |
| Control  Footshock  No Footshock | 28.89 ± 13.52  7.14 ± 4.63  18.09 ± 9.7 |
