# Supplementary material for: Shifting from fear to safety through deconditioning-update
Source: eLife. 2020 Jan 30;9:e51207. doi: 10.7554/eLife.51207 (PMC7021486; doi:10.7554/eLife.51207)
Supplement: Supplementary file 13. [file elife-51207-supp13.docx]

**Table 13. Baseline (pre-CS freezing levels for Figure 2.**

| Figure 2 | |
| --- | --- |
| Reactivations | |
| Group | Baseline (% ± SEM) |
| Day 41  No Footshock  Footshock  Day 42  No Footshock  Footshock  Day 43  No Footshock  Footshock  Day 44  No Footshock  Footshock | 89.52 ± 4.26  85 ± 5.28  75.71 ± 8.15  13.33 ± 6.09  37.62 ± 10.38  28.89 ± 11.47  59.05 ± 11.81  10 ± 5.96 |
| Test | |
| Group | Baseline (% ± SEM) |
| Control  Footshock  No Footshock | 91.33 ± 4.29  12.22 ± 9.65  7.62 ± 3.4 |
| Renewal | |
| Group | Baseline (% ± SEM) |
| Control  Footshock  No Footshock | 86.67 ± 3.16  2.22 ± 1.65  19.52 ± 12.98 |
| Spontaneous Recovery | |
| Group | Baseline (% ± SEM) |
| Control  Footshock  No Footshock | 49.33 ± 16.58  8.33 ± 6.07  33.89 ± 13.44 |
| Reactivations | |
| Group | Baseline (% ± SEM) |
| Day 3  No Footshock  Footshock  Day 4  No Footshock  Footshock  Day 5  No Footshock  Footshock  Day 6  No Footshock  Footshock | 71.43 ± 6.97  76.67 ± 5.59  59.05 ± 9.19  42.86 ± 11.44  32.38 ± 10.01  29.52 ± 9.18  20.95 ± 10.61  23.81 ± 12.59 |
| Test | |
| Group | Baseline (% ± SEM) |
| Control  Footshock  No Footshock | 59.45 ± 18.02  16.19 ± 10.5  6.19 ± 4.74 |
| Renewal | |
| Group | Baseline (% ± SEM) |
| Control  Footshock  No Footshock | 66.11 ± 14.81  17.62 ± 10.86  23.81 ± 7.47 |
| Spontaneous Recovery | |
| Group | Baseline (% ± SEM) |
| Control  Footshock  No Footshock | 61.11 ± 12.18  10.48 ± 9.41  44.76 ± 11.77 |
