# Supplementary material for: Shifting from fear to safety through deconditioning-update
Source: eLife. 2020 Jan 30;9:e51207. doi: 10.7554/eLife.51207 (PMC7021486; doi:10.7554/eLife.51207)
Supplement: Supplementary file 14. [file elife-51207-supp14.docx]

**Table 14. Baseline (pre-CS) freezing levels for Figure 4.**

| Figure 4 | |
| --- | --- |
| Extinction Sessions | |
| Group | Baseline (% ± SEM) |
| No Footshock  Footshock | 27.62 ± 14.3  31.9 ± 14.74 |
| Test | |
| Group | Baseline (% ± SEM) |
| Control  Footshock  No Footshock | 49.44 ± 17.48  51.9 ± 14.69  10.95 ± 7 |
| Renewal | |
| Group | Baseline (% ± SEM) |
| Control  Footshock  No Footshock | 68.33 ± 10.81  56.67 ± 11.1  48.57 ± 13.68 |
| Spontaneous Recovery | |
| Group | Baseline (% ± SEM) |
| Control  Footshock  No Footshock | 70.56 ± 5.12  31.43 ± 15.17  10.95 ± 6.62 |
| Reactivations | |
| Group | Baseline (% ± SEM) |
| Day 3  No Footshock + Vehicle  No Footshock + Nimodipine  Footshock + Vehicle  Footshock + Nimodipine  Day 4  No Footshock + Vehicle  No Footshock + Nimodipine  Footshock + Vehicle  Footshock + Nimodipine  Day 5  No Footshock + Vehicle  No Footshock + Nimodipine  Footshock + Vehicle  Footshock + Nimodipine  Day 6  No Footshock + Vehicle  No Footshock + Nimodipine  Footshock + Vehicle  Footshock + Nimodipine | 60.95 ± 14.47  68.57 ± 9.22  44.29 ± 10.25  55.71 ± 10.77  47.62 ± 10.9  44.29 ± 11.97  36.19 ± 9.04  47.14 ± 14.34  43.81 ± 8.69  27.62 ± 7.48  16.19 ± 7.98  46.19 ± 13.73  17.14 ± 10.06  32.86 ± 12.73  11.43 ± 8.22  49.17 ± 14.44 |
| Test | |
| Group | Baseline (% ± SEM) |
| No Footshock + Vehicle  No Footshock + Nimodipine  Footshock + Vehicle  Footshock + Nimodipine | 29.05 ± 12.55  21.91 ± 10.79  1.42 ± 1.142  35 ± 12.18 |
| Renewal | |
| Group | Baseline (% ± SEM) |
| No Footshock + Vehicle  No Footshock + Nimodipine  Footshock + Vehicle  Footshock + Nimodipine | 42.86 ± 12.63  10 ± 4.93  17.14 ± 736  40.83 ± 11.84 |
| Spontaneous Recovery | |
| Group | Baseline (% ± SEM) |
| No Footshock + Vehicle  No Footshock + Nimodipine  Footshock + Vehicle  Footshock + Nimodipine | 36.67 ± 9.54  14.76 ± 8.63  18.57 ± 7.96  32 ± 9.48 |
