# Supplementary material for: Shifting from fear to safety through deconditioning-update
Source: eLife. 2020 Jan 30;9:e51207. doi: 10.7554/eLife.51207 (PMC7021486; doi:10.7554/eLife.51207)
Supplement: Supplementary file 15. [file elife-51207-supp15.docx]

**Table 15. Baseline (pre-CS) freezing levels for Figure 1-figure supplement 1.**

| Figure 1S1 | |
| --- | --- |
| Reactivations | |
| Group | Baseline (% ± SEM) |
| Day 3  No Footshock  Footshock  Day 4  No Footshock  Footshock  Day 5  No Footshock  Footshock  Day 6  No Footshock  Footshock | 42.38 ± 14.23  39.05 ± 12.82  29.05 ± 11.22  43.33 ± 9.97  21.43 ± 12.7  56.67 ± 13.72  8.09 ± 6.58  25.24 ± 11.17 |
| Test | |
| Group | Baseline (% ± SEM) |
| Control  Footshock  No Footshock | 76.11 ± 15.16  49.52 ± 14.34  11.9 ± 8.35 |
