# Supplementary material for: Shifting from fear to safety through deconditioning-update
Source: eLife. 2020 Jan 30;9:e51207. doi: 10.7554/eLife.51207 (PMC7021486; doi:10.7554/eLife.51207)
Supplement: Supplementary file 16. [file elife-51207-supp16.docx]

**Table 16. Baseline (pre-CS) freezing levels for Figure -figure supplement 2.**

| Figure 1S2 | |
| --- | --- |
| Reactivations | |
| Group | Baseline (% ± SEM) |
| Day 3  No Footshock  Footshock | 70.95 ± 13.64  59.05 ± 12.25 |
| Test | |
| Group | Baseline (% ± SEM) |
| Control  Footshock  No Footshock | 78.33 ± 9.06  60.48 ± 10.48  68.57 ± 11.12 |
| Renewal | |
| Group | Baseline (% ± SEM) |
| Control  Footshock  No Footshock | 70.56 ± 13.59  49.52 ± 9.58  52.38 ± 13.94 |
