# Supplementary material for: Shifting from fear to safety through deconditioning-update
Source: eLife. 2020 Jan 30;9:e51207. doi: 10.7554/eLife.51207 (PMC7021486; doi:10.7554/eLife.51207)
Supplement: Supplementary file 17. [file elife-51207-supp17.docx]

**Table S17. Baseline (pre-CS) freezing levels for Figure 1-figure supplement 3.**

| Figure 1S3 | |
| --- | --- |
| Test | |
| Group | Baseline (% ± SEM) |
| Control  Footshock  No Footshock | 94.29 ± 1.88  26.67 ± 12.08  47.14 ± 16.75 |
| Reactivations | |
| Group | Baseline (% ± SEM) |
| Day 3  No Footshock  Footshock  Day 4  No Footshock  Footshock  Day 5  No Footshock  Footshock  Day 6  No Footshock  Footshock | 64.33 ± 11.85  72.33 ± 11.32  51.67 ± 9.27  52.67 ± 11.88  36.67 ± 9.5  29.33 ± 14.13  26.33 ± 11.51  25.33 ± 13.06 |
| Test | |
| Group | Baseline (% ± SEM) |
| No Footshock  Footshock | 35.19 ± 13.51  9.26 ± 4.82 |
| Reinstatement | |
| Group | Baseline (% ± SEM) |
| No Footshock  Footshock | 6.25 ± 3.67  0 ± 0 |
