# Supplementary material for: Shifting from fear to safety through deconditioning-update
Source: eLife. 2020 Jan 30;9:e51207. doi: 10.7554/eLife.51207 (PMC7021486; doi:10.7554/eLife.51207)
Supplement: Supplementary file 18. [file elife-51207-supp18.docx]

**Table 18. Baseline (pre-CS) freezing levels for Figure 1-figure supplement 4.**

| Figure 1S4 | |
| --- | --- |
| Reactivations | |
| Group | Baseline (% ± SEM) |
| Day 3  Footshock  Footshock unpaired  Day 4  Footshock  Footshock unpaired  Day 5  Footshock  Footshock unpaired  Day 6  Footshock  Footshock unpaired | 45 ± 13.83  48.33 ± 8.45  47.08 ± 12.61  47.92 ± 5.63  10.83 ± 5.9  10.42 ± 4.38  4.17 ± 4.17  18.33 ± 8.61 |
| Test | |
| Group | Baseline (% ± SEM) |
| Footshock  Footshock unpaired | 4.17 ± 3  14.17 ± 7.2 |
