# Supplementary material for: Shifting from fear to safety through deconditioning-update
Source: eLife. 2020 Jan 30;9:e51207. doi: 10.7554/eLife.51207 (PMC7021486; doi:10.7554/eLife.51207)
Supplement: Supplementary file 19. [file elife-51207-supp19.docx]

**Table 19. Baseline (pre-CS) freezing levels for Figure 2-supplement 1.**

| Figure 2S1 | |
| --- | --- |
| Reactivations | |
| Group | Baseline (% ± SEM) |
| Day 3  No Footshock  Footshock  Day 4  No Footshock  Footshock  Day 5  No Footshock  Footshock | 29.05 ± 10.78  24.44 ± 10.35  37.14 ± 11.51  34.44 ± 10.56  37.62 ± 13.41  18.89 ± 10.13 |
| Test | |
| Group | Baseline (% ± SEM) |
| Control  Footshock  No Footshock | 23.33 ± 15.35  5 ± 3.82  21.91 ± 10.59 |
| Renewal | |
| Group | Baseline (% ± SEM) |
| Control  Footshock  No Footshock | 36.11 ± 15.07  3.89 ± 3.88  30.95 ± 13.39 |
