# Supplementary material for: Shifting from fear to safety through deconditioning-update
Source: eLife. 2020 Jan 30;9:e51207. doi: 10.7554/eLife.51207 (PMC7021486; doi:10.7554/eLife.51207)
Supplement: Supplementary file 20. [file elife-51207-supp20.docx]

**Table 20. Baseline (pre-CS) freezing levels for Figure 4-figure supplement 1.**

| Figure 4S1 | |
| --- | --- |
| Extinction Session | |
| Group | Baseline (% ± SEM) |
| No Footshock  Footshock | 39.05 ± 16.42  52.38 ± 17.95 |
| Test | |
| Group | Baseline (% ± SEM) |
| Control  Footshock  No Footshock | 41.11 ± 17.44  30.48 ± 15.76  40 ± 17.15 |
| Renewal | |
| Group | Baseline (% ± SEM) |
| Control  Footshock  No Footshock | 46.67 ± 13.31  12.86 ± 9.55  19.05 ± 12.29 |
| Spontaneous Recovery | |
| Group | Baseline (% ± SEM) |
| Control  Footshock  No Footshock | 47.78 ± 17.65  41.91 ± 14.87  52.86 ± 11.47 |
