# Supplementary material for: Shifting from fear to safety through deconditioning-update
Source: eLife. 2020 Jan 30;9:e51207. doi: 10.7554/eLife.51207 (PMC7021486; doi:10.7554/eLife.51207)
Supplement: Supplementary file 21. [file elife-51207-supp21.docx]

**Table 21. Baseline (pre-CS) freezing levels for Figure 4-figure supplement 2.**

| Figure 4S2 | |
| --- | --- |
| Reactivations | |
| Group | Baseline (% ± SEM) |
| Day 3  Footshock + Vehicle  Footshock + Nimodipine  Day 4  Footshock + Vehicle  Footshock + Nimodipine  Day 5  Footshock + Vehicle  Footshock + Nimodipine  Day 6  Footshock + Vehicle  Footshock + Nimodipine | 80.56 ± 6.11  52.86 ± 9.93  71.67 ± 7.59  74.29 ± 4.13  50.56 ± 9.37  53.81 ± 6.95  27.78 ± 7.49  21.43 ± 6.14 |
| Test 1 | |
| Group | Baseline (% ± SEM) |
| Footshock + Vehicle  Footshock + Nimodipine | 7.22 ± 5.47  18.09 ± 4.49 |
| Test 2 | |
| Group | Baseline (% ± SEM) |
| Footshock + Vehicle  Footshock + Nimodipine | 5.56 ± 5.56  10 ± 3.78 |
