# Supplementary material for: Shifting from fear to safety through deconditioning-update
Source: eLife. 2020 Jan 30;9:e51207. doi: 10.7554/eLife.51207 (PMC7021486; doi:10.7554/eLife.51207)
Supplement: Peer review history

# Decision letter

**DOI:** 10.7554/eLife.51207.sa1

**Affiliations:** University of MarylandUnited States; NYU Langone HealthUnited States; Texas A&M UniversityUnited States; University of Texas at AustinUnited States

In the interests of transparency, eLife publishes the most substantive revision requests and the accompanying author responses.

**Acceptance summary:**

Popik and colleagues report a series of "deconditioning" experiments in rats. In deconditioning, rats are re-exposed to Pavlovian fear conditioning trials with a much weaker shock over 4 consecutive days. Subsequent fear expression is dramatically reduced compared to box- and extinction-controls – presumably via a reconsolidation/memory updating process that alters the underlying CS-US association. Unlike extinction, this reduction is resistant to renewal and spontaneous recovery. Deconditioning also works with context fear conditioning and inhibitory avoidance, and it does not appear to be constrained by known reconsolidation boundary conditions (i.e. very strong or old memories are still susceptible). The authors also demonstrate that L-type voltage-gated calcium channel blockers – which have been shown to be necessary for extinction and memory destabilization – prevent deconditioning. This is an interesting and important addition to the reconsolidation/memory updating literature that reports an innovative approach for modifying fear memories. The manuscript is well-written. The basic effect is replicated several times for both males and female subjects. The paper is strengthened by behavioral pharmacology and modeling data.

**Decision letter after peer review:**

Thank you for submitting your article "Shifting from fear to safety through deconditioning-update: a novel approach to attenuate fear memories" for consideration by *eLife*. Your article has been reviewed by a Senior Editor (Kate Wassum), a Reviewing Editor (Alex Shackman), and three reviewers. The following individuals involved in review of your submission have agreed to reveal their identity: Christopher Cain (Reviewer #1); Travis D. Goode (Reviewer #2); Marie H Monfils (Reviewer #3).

The reviewers and the Reviewing Editor have discussed the reviews with one another and the Reviewing Editor has drafted this decision to help you prepare a revised submission.

Essential revisions:

1) If the authors wish to keep the Nimodipine experiment in the manuscript, it's essential that they add a state-dependent control group. The nimodipine experiments lack an important control group that is necessary to conclude that the drug blocks deconsolidation. See Figure 4F-J. With systemic drugs it is essential to demonstrate that memory impairments persist when the animals are tested in the drugged-condition, in case the drug state becomes part of the memory and is a necessary stimulus condition for retrieval. This is not just theoretical Bouton and others have shown apparent extinction impairments (e.g. with benzos) that go away if the subjects are tested in the drugged condition.

2) One of the reviewers questioned whether arousal is a confounding factor, and wondered whether it would be useful to test whether animals exposed to non-contingent shocks show deconditioning effects: "A critical finding in the manuscript is that these effects of deconditioning appear dependent on multiple short reactivation sessions (as opposed to a single massed session where such a deconditioning procedure actually impedes extinction; Figure 4—figure supplement 1 shows similar weak effects). As such (and given the high levels of freezing in footshock animals undergoing massed extinction) I'm wondering if stress/arousal levels (instead of, or in addition to, mismatch signals) may be a dominant factor here (given that animals are nevertheless receiving shocks). In other words, weak (but aversive) footshock presentations (in addition to the fear-eliciting CS) may enhance stress responding (norepinephrine-release, for example) which may facilitate consolidation/reconsolidation of the forming extinction memory (perhaps to a greater extent than in the no footshock groups). In the case of the massed extinction, this stress responding may not be optimal to facilitate extinction given the mass of trials (and perhaps new sensitization/conditioning). To this end, a critical experiment may be to add a group of animals to receive non-contingent presentations of the low-level shocks (perhaps randomly in the inter-CS intervals) to see whether the same "deconditioning"-like effects can be induced by shock-induced arousal that is concomitant with CS-elicited arousal and reactivation/extinction (but not linked to mismatch per se since it does not occur at the end of the tone). If similar deconditioning effects are seen with unpaired shocks, then these effects may mirror post-training stress-/arousal-dependent facilitation of learning seen in other tasks. If this fails to produce the deconditioning-like effects, then this would further support the extinction-promoting effects of graded prediction error/mismatch."

There was some discussion among reviewers on this point, and they agreed that they are comfortable letting the authors address this concern either through revisions to the text or through a new experiment.

[Editors' note: further revisions were suggested prior to acceptance, as described below.]

Thank you for resubmitting your work entitled "Shifting from fear to safety through deconditioning-update" for further consideration by *eLife*. Your revised article has been evaluated by Kate Wassum (Senior Editor) and Alex Shackman (Reviewing Editor).

The manuscript has been greatly improved but there are some remaining issues that need to be addressed before acceptance, as outlined below:

1) In the Materials and methods section, indicate that pre-CS freezing was also scored and describe how (e.g., for an equivalent duration as the CS or the whole acclimation period).

2) In the Results section, please report the pattern of pre-CS freezing and direct the reader to the actual data (Supplementary files).

3) In the Discussion section, please offer some interpretation/explanation for the high pre-CS freezing and why this does not undermine confidence in the main findings.

(As the reviewer noted – "Baseline freezing during the reactivation and test sessions is often very high (sometimes greater than 70%) and a bit erratic. I suspect this is because the different contexts didn't actually differ that much leading to generalization. However, after plotting these against CS-freezing data for a few of the experiments it doesn't appear to substantially change the pattern of results. I considered asking that pre-CS freezing be included in at least the test graphs -- but I think this could make the figures more cluttered and diminish understanding of the main effects. I don't think it's enough to simply include dozens of supplemental tables and ignore the issue in the main text.")

4) Authors state that "Lower mismatch accelerates extinction and decreases renewal in a neural network model." A reviewer noted that they remain unconvinced that the data support that claim, nothing that, "I don't believe extinction is tested directly in the referenced experiment, since it is assessed across days. As such, it is impossible to evaluate whether the decrease in "CR" is evidence of accelerated/facilitated extinction, reconciliation blockade, facilitated extinction consolidation, or some other possible explanation. In my view, acceleration of extinction implies a more rapid within session facilitation of extinction." Authors should revise the manuscript to address/acknowledge this perspective on the data.

5) Monfils et al., 2009 should still appear in the introduction, since it was one of the first to employ a reconsolidation "updating" manipulation and, hence, is quite relevant to the present work.

6) Clarify how the effect size was estimated for the power analyses. How did the authors arrive at 90% power?

# Reply: Author response

**DOI:** 10.7554/eLife.51207.sa2

Summary:

Popik and colleagues report a series of "deconditioning" experiments in rats. In deconditioning, rats are re-exposed to Pavlovian fear conditioning trials with a much weaker shock over 4 consecutive days. Subsequent fear expression is dramatically reduced compared to box- and extinction-controls – presumably via a reconsolidation/memory updating process that alters the underlying CS-US association. Unlike extinction, this reduction is resistant to renewal and spontaneous recovery. Deconditioning also works with context fear conditioning and inhibitory avoidance, and it does not appear to be constrained by known reconsolidation boundary conditions (i.e. very strong or old memories are still susceptible). Authors also demonstrate that L-type voltage-gated calcium channel blockers – which have been shown to be necessary for extinction and memory destabilization – prevent deconditioning. This is an interesting and important addition to the reconsolidation/memory updating literature that reports an innovative approach for modifying fear memories. The manuscript is well-written. The basic effect is replicated several times for both males and female subjects. The paper is strengthened by behavioral pharmacology and modeling data.

Essential revisions:

1) If the authors wish to keep the Nimodipine experiment in the manuscript, it's essential that they add a state-dependent control group. The nimodipine experiments lack an important control group that is necessary to conclude that the drug blocks deconsolidation. See Figure 4F-J. With systemic drugs it is essential to demonstrate that memory impairments persist when the animals are tested in the drugged-condition, in case the drug state becomes part of the memory and is a necessary stimulus condition for retrieval. This is not just theoretical Bouton and others have shown apparent extinction impairments (e.g. with benzos) that go away if the subjects are tested in the drugged condition.

We thank the reviewers for bringing this important point to our attention. As suggested, we performed an additional experiment in order to address this issue. We trained 20 animals and divided them in two groups. The treated group received nimodipine 30 minutes before each reactivation, while the control group received vehicle. In the test, animals in the treated group received either vehicle or nimodipine. If the nimodipine treatment was making this memory state-dependent, then we would expect that drug injection before the test would decrease the freezing response. Our results show that nimodipine injection during reactivation does not induce a state-dependent memory, since the performance in the test with or without nimodipine is the same. We thus suggest that the nimodipine treatment before reactivation is acting primarily on the memory destabilization, preventing deconditioning-update.

We now refer to this experiment in the Results section and present the results in Figure 4—figure supplement 2.

2) One of the reviewers questioned whether arousal is a confounding factor, and wondered whether it would be useful to test whether animals exposed to non-contingent shocks show deconditioning effects: "A critical finding in the manuscript is that these effects of deconditioning appear dependent on multiple short reactivation sessions (as opposed to a single massed session where such a deconditioning procedure actually impedes extinction; Figure 4—figure supplement 1 shows similar weak effects). As such (and given the high levels of freezing in footshock animals undergoing massed extinction) I'm wondering if stress/arousal levels (instead of, or in addition to, mismatch signals) may be a dominant factor here (given that animals are nevertheless receiving shocks). In other words, weak (but aversive) footshock presentations (in addition to the fear-eliciting CS) may enhance stress responding (norepinephrine-release, for example) which may facilitate consolidation/reconsolidation of the forming extinction memory (perhaps to a greater extent than in the no footshock groups). In the case of the massed extinction, this stress responding may not be optimal to facilitate extinction given the mass of trials (and perhaps new sensitization/conditioning). To this end, a critical experiment may be to add a group of animals to receive non-contingent presentations of the low-level shocks (perhaps randomly in the inter-CS intervals) to see whether the same "deconditioning"-like effects can be induced by shock-induced arousal that is concomitant with CS-elicited arousal and reactivation/extinction (but not linked to mismatch per se since it does not occur at the end of the tone). If similar deconditioning effects are seen with unpaired shocks, then these effects may mirror post-training stress-/arousal-dependent facilitation of learning seen in other tasks. If this fails to produce the deconditioning-like effects, then this would further support the extinction-promoting effects of graded prediction error/mismatch."

There was some discussion among reviewers on this point, and they agreed that they are comfortable letting the authors address this concern either through revisions to the text or through a new experiment.

We agree with the reviewers that it is an important point to be explored and have thus performed a new experiment in order to test this possible confounding factor. We conditioned animals as described in the Figure 1A, but in the reactivation sessions, the CS and the US were presented in an unpaired manner. We found that if the weak US is not presented contingent with the CS, there is no fear reduction in the test, leading to significantly greater freezing than in the deconditioning-update group.

We refer to this experiment in the Results section and show its results in Figure 1—figure supplement 4.

[Editors' note: further revisions were suggested prior to acceptance, as described below.]

The manuscript has been greatly improved but there are some remaining issues that need to be addressed before acceptance, as outlined below:

1) In the Materials and methods section, indicate that pre-CS freezing was also scored and describe how (e.g., for an equivalent duration as the CS or the whole acclimation period).

Pre-CS freezing was measured for the 30 seconds immediately preceding the first tone (i.e. a duration equivalent to that of the tone). We have now included this information in the Materials and methods section of the manuscript. Note that these values are typically higher than measurements taken over the full 2 minutes before the tone, as freezing in the first minute tends to be lower.

2) In the Results section, please report the pattern of pre-CS freezing and direct the reader to the actual data (Supplementary files).

As suggested, we have reported this fact in the results (when discussing Figure 1) and included a direct reference to the tables regarding the corresponding pre-CS information in each figure legend.

3) In the Discussion section, please offer some interpretation/explanation for the high pre-CS freezing and why this does not undermine confidence in the main findings.

(As the reviewer noted – "Baseline freezing during the reactivation and test sessions is often very high (sometimes greater than 70%) and a bit erratic. I suspect this is because the different contexts didn't actually differ that much leading to generalization. However, after plotting these against CS-freezing data for a few of the experiments it doesn't appear to substantially change the pattern of results. I considered asking that pre-CS freezing be included in at least the test graphs -- but I think this could make the figures more cluttered and diminish understanding of the main effects. I don't think it's enough to simply include dozens of supplemental tables and ignore the issue in the main text.")

We agree with the reviewer that some degree of generalization is indeed occurring, as many animals express a high freezing level even before the tone, probably due to the similarity between the contexts A and B. Nevertheless, we note that switching the animals back to context A after extinction (i.e. retrieval) does lead to increased freezing levels – thus, at least some degree of differentiation between contexts is maintained. We agree with the reviewer that this issue should be discussed in the main text, and we now address it in the Discussion section, along with its implications for the deconditioning-update effect.

We also thought about including pre-CS freezing in the figures, but it would be rather complicated to do so, as each session would require its own pre-CS value. Thus, we agree with the reviewer that it would probably cause unnecessary confusion and hamper understanding the main effects (e.g. the differences in freezing in response to the tone) and have chosen not to do it.

4) Authors state that "Lower mismatch accelerates extinction and decreases renewal in a neural network model." A reviewer noted that they remain unconvinced that the data support that claim, nothing that, "I don't believe extinction is tested directly in the referenced experiment, since it is assessed across days. As such, it is impossible to evaluate whether the decrease in "CR" is evidence of accelerated/facilitated extinction, reconciliation blockade, facilitated extinction consolidation, or some other possible explanation. In my view, acceleration of extinction implies a more rapid within session facilitation of extinction." Authors should revise the manuscript to address/acknowledge this perspective on the data.

We agree with the reviewer that referring to our findings as “accelerated extinction” is misleading, as there is no way to clearly differentiate accelerated extinction from reconsolidation-blockade effects in terms of behavior alone: we had referred to “extinction” in the strictly behavioral sense in this case, but it is indeed not in line with what the model itself predicts. We have thus changed the statement “accelerate extinction” to “accelerates fear reduction” both in the Discussion section and in the legend of Figure 5.

5) Monfils et al., 2009 should still appear in the introduction, since it was one of the first to employ a reconsolidation "updating" manipulation and, hence, is quite relevant to the present work.

We agree with the reviewer, and we have inserted this reference twice in the Introduction.

6) Clarify how the effect size was estimated for the power analyses. How did the authors arrive at 90% power?

We do not clearly understand what is meant by ‘estimated’, as effect sizes used for power calculations are usually chosen based on biological significance (e.g. a minimum effect size of interest) rather than through estimation of the effect size will actually be (although pilot data or other studies can be used for these means). We considered a 30% decrease in absolute freezing as a meaningful difference between reactivation procedures – on a range of that between the 1h retrieval-extinction groups with the mean of those outside the reconsolidation window in Monfils et al., 2009, for example.

Calculations were originally performed using the parameters mentioned in the manuscript (i.e. a 30% difference in freezing time, a standard deviation of 15%, α = 0.05, sample sizes between 6 and 10) for a 2-tailed t test using G*Power 3.1). Power in this case ranges from 90% in the worst-case scenario in our article (comparisons between groups with n = 6 and 7, respectively) to 99% in the best-case scenario (comparisons between groups with n = 10).

Nevertheless, in revising the calculations we have noticed that using 15% may have been an underestimation of the standard deviation in our experiments, as our variation was on average slightly higher. For Figure 1, for example, the average standard deviation in the Footshock and No-Footshock groups was around 18% for all sessions and 20% for the test sessions (i.e. excluding the reactivation ones, which begin with very low standard deviations).

We have thus chosen to revise the calculation included in the manuscript to account for a 20% standard deviation. This leads power to range between 69% in the worst-case scenario and 89% in the best-case one. The Materials and methods section has been revised accordingly, including a reference to Monfils et al., 2009 for the effect size used.
